# Efficient Autotransporter-Mediated Extracellular Secretion of a Heterologous Recombinant Protein by Escherichia coli

**DOI:** 10.1128/spectrum.03594-22

**Published:** 2023-04-10

**Authors:** Irene Beriotto, Christopher Icke, Yanina R. Sevastsyanovich, Amanda E. Rossiter, Giacomo Romagnoli, Silvana Savino, Freya J. Hodges, Jeffrey A. Cole, Allan Saul, Calman A. MacLennan, Adam F. Cunningham, Francesca Micoli, Ian R. Henderson

**Affiliations:** a Institute of Microbiology and Infection, University of Birmingham, Birmingham, United Kingdom; b GSK Vaccines Institute for Global Health Srl, Siena, Italy; c Institute for Molecular Bioscience, University of Queensland, Brisbane, Queensland, Australia; d Jenner Institute, University of Oxford, Oxford, United Kingdom; e Institute for Immunology and Immunotherapy, University of Birmingham, Birmingham, United Kingdom; Cinvestav-IPN

**Keywords:** autotransporter, *Escherichia coli*, recombinant protein production, secretion, inclusion bodies, Pet autotransporter platform, autotransporter proteins, protein secretion

## Abstract

The autotransporter protein secretion system has been used previously to target the secretion of heterologous proteins to the bacterial cell surface and the extracellular milieu at the laboratory scale. The platform is of particular interest for the production of “difficult” recombinant proteins that might cause toxic effects when produced intracellularly. One such protein is IrmA. IrmA is a vaccine candidate that is produced in inclusion bodies requiring refolding. Here, we describe the use and scale-up of the autotransporter system for the secretion of an industrially relevant protein (IrmA). A plasmid expressing IrmA was constructed such that the autotransporter platform could secrete IrmA into the culture supernatant fraction. The autotransporter platform was suitable for the production and purification of IrmA with comparable physical properties to the protein produced in the cytoplasm. The production of IrmA was translated to scale-up protein production conditions resulting in a yield of 29.3 mg/L of IrmA from the culture supernatant, which is consistent with yields of current industrial processes.

**IMPORTANCE** Recombinant protein production is an essential component of the biotechnology sector. Here, we show that the autotransporter platform is a viable method for the recombinant production, secretion, and purification of a “difficult” to produce protein on an industrially relevant scale. Use of the autotransporter platform could reduce the number of downstream processing operations required, thus accelerating the development time and reducing costs for recombinant protein production.

## INTRODUCTION

Recombinant protein production is a cornerstone of the molecular biology revolution and the biotechnology industry. Escherichia coli is used frequently to produce proteins (1, 2). However, many of these proteins are produced in the cytoplasm of E. coli and often aggregate into inclusion bodies, requiring multiple downstream processing steps to achieve a soluble, pure, functional protein and thereby increasing production costs and time to market. In contrast, the accumulation of the target protein in the culture supernatant fraction could reduce downstream processing steps and production time, resulting in significant cost savings (3). Many bacterial secretion systems have been harnessed to deliver recombinant heterologous proteins into the culture supernatant (4). One such system that has been well studied, which is attractive because of its simplicity, is the classical autotransporter (AT) secretion system. This system is found widely among most Gram-negative bacterial species (5–7). These AT proteins are a subset of the type 5 secretion system (T5SS) and typically include an N-terminal signal sequence that mediates translocation across the cytoplasmic membrane, a functionally discrete passenger, an autochaperone (AC) domain that assists in folding of the passenger, and a C-terminal β-barrel domain that facilitates the insertion of the protein into the outer membrane by the BAM complex and subsequent secretion of the passenger across the bacterial outer membrane (6, 8–11). By replacement of the passenger with a protein of interest, the AT system can be engineered such that the protein of interest can be displayed on the bacterial cell surface or released into the extracellular milieu (3, 12, 13).

The AT platform offers an alternative to other traditional systems for cytoplasmic expression and may offer a solution to produce “difficult proteins” that are toxic or misfolded when retained within the cell. Particularly attractive is that the protein is secreted extracellularly. Extracellular secretion could enable toxic protein production to be maintained by bacteria for longer than traditional systems. Additionally, protein can accumulate in the medium at high concentrations. However, while the AT platform can accumulate heterologous proteins in the culture medium at laboratory scale, there is no evidence to support the utility of the AT platform for recombinant protein production at scales useful for industrial protein production. Consequently, we sought to produce interleukin receptor mimic protein A (IrmA) to test the efficacy of the AT platform for the production of heterologous proteins in an industrial setting.

**FIG 1:**
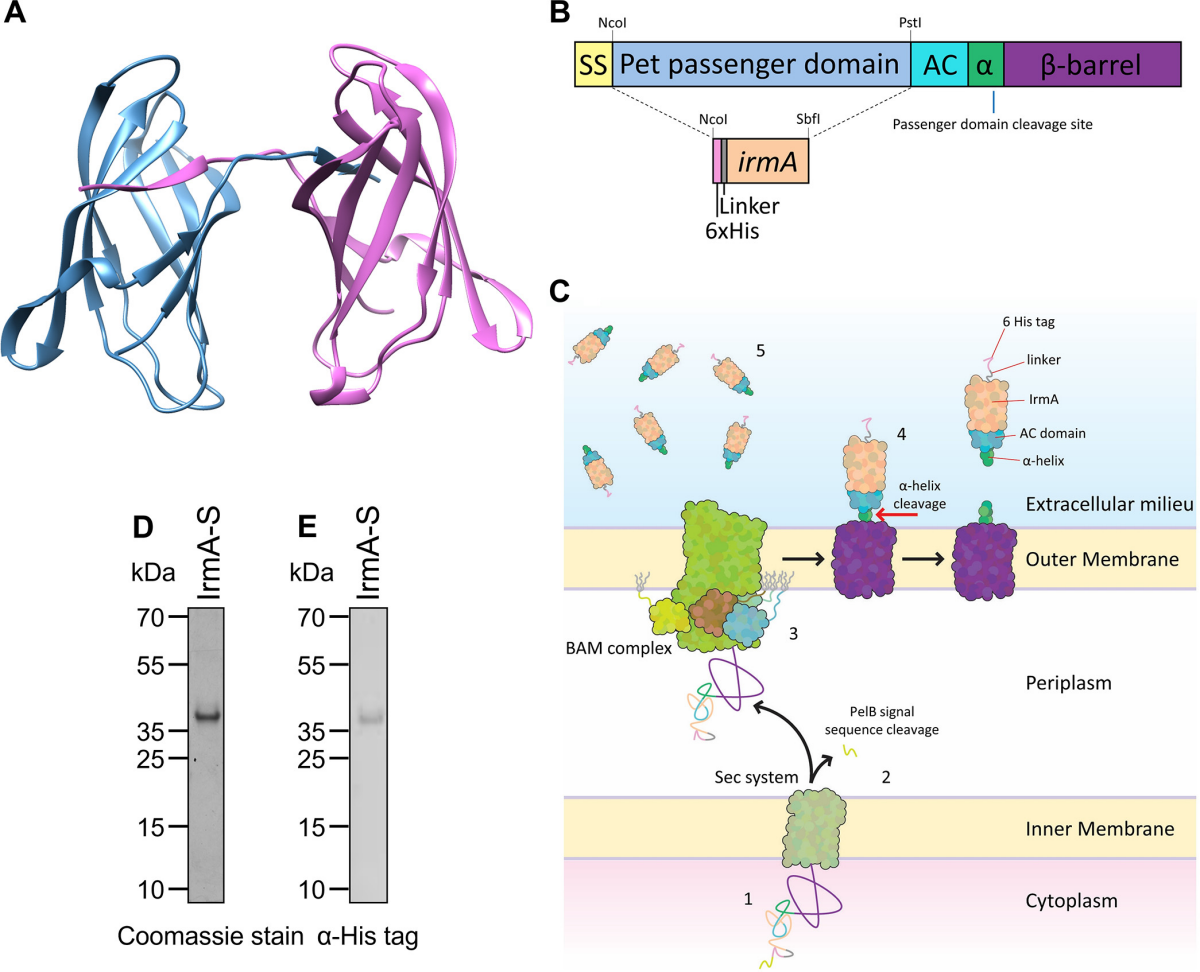
IrmA-S construct and secretion from E. coli BL21. (A) Crystal structure of the IrmA dimer (retrieved from PDB 5EK5). (B) Schematic diagram of the IrmA-S construct, not to scale. The *irmA* gene is inserted into the Pet AT platform, replacing the native Pet passenger and leaving a *pelB* signal sequence (SS), a 6× His tag, a linker region, the *irmA* gene, the autochaperone domain (AC), and the α-helix (α), the site at which the passenger is cleaved from the β-barrel. The plasmid encoding this construct was termed pET22b-IrmA-S. (C) Secretion of IrmA-S by the AT platform occurs in 5 steps, which are labeled as follows: 1, the AT platform is produced in the cytoplasm; 2, the AT platform is secreted into the periplasm by the Sec system and the PelB signal sequence is cleaved; 3, the AT platform is inserted into the outer membrane by the BAM complex; 4, the folded AT platform undergoes autoproteolysis releasing IrmA-S; and 5, IrmA-S accumulates in the culture medium. (D) The culture supernatant of E. coli BL21*DE3 *dsbA*::*aph* harboring the plasmid pET22b-IrmA-S after IPTG induction was analyzed for the presence of IrmA-S by SDS-PAGE and stained with Coomassie blue. (E) The same sample was subjected to immunoblotting using α-His antibodies. Note that although the theoretical molecular mass of IrmA-S is 29.8 kDa, it migrated in the gel with a higher apparent molecular mass.

IrmA (16.9 kDa) is a secreted homodimeric protein, where each protomer contains an incomplete fibronectin III (FNIII)-like domain (Fig. 1A) (14). Characteristic of the FNIII-like domains, IrmA is composed of two aligned β-sheets, forming a β-sandwich fold (14). The dimer structure is formed by a unique domain swap mechanism where the C-terminal unstructured region and β-strand from each protomer complete the FNIII-like fold of the other (14). It is produced by uropathogenic Escherichia coli (UPEC), a causative agent of urinary tract infections (UTIs), and some strains of diarrheagenic E. coli (14, 15). UTIs affect over half of all women during their lifetime with the associated antimicrobial treatment accounting for 15% of outpatient prescriptions (16). IrmA is a putative vaccine candidate that when used in immunization studies can induce protection from challenge with UPEC (17). Currently, IrmA for use as a vaccine is produced in E. coli in inclusion bodies and is purified from the cytoplasmic fraction using a pre-established proprietary methodology that includes protein refolding, affinity chromatography, sample desalting, ion-exchange chromatography, and finally a dialysis step (we have designated this form of the protein IrmA-C). However, the complexities of producing IrmA and the low protein yields from scaled up production have hampered the use of IrmA for vaccine studies, and as a result, IrmA has been classified as a “difficult protein” for recombinant protein production. Thus, we sought to investigate if the use of the AT platform would alleviate the limitations of IrmA production.

Previously, we have investigated the use of the Pet AT as a platform for the secretion of heterologous proteins (3). Pet is a member of the serine protease ATs of the *Enterobacteriaceae* (SPATEs), and as such, it is autocatalytic. This autocatalytic activity liberates the passenger from the AT enabling direct secretion into the extracellular environment (3). Here, we investigated whether the Pet AT platform could be used to produce and secrete IrmA into the culture medium (IrmA-S) at an industrial scale, bypassing the limitations of the cytosolic production of IrmA, and thus reducing the complexity of its production process, saving time, and saving costs.

## RESULTS

### Production of secreted IrmA-S.

Previously, we described an AT protein production platform based on the Pet serine protease of E. coli. We demonstrated that the Pet AT platform could secrete a variety of heterologous proteins to the bacterial cell surface or into the extracellular milieu (3). IrmA was selected to test whether the Pet AT platform could be exploited to produce a “difficult” protein with commercial relevance. The *irmA* open reading frame without a stop codon was cloned into a Novagen vector, pET22b, containing the *pet* AT sequence. Our previous work demonstrated that the efficiency of the secretion of heterologous passengers was diminished on truncation of the AC domain (3). Therefore, the *irmA* gene was cloned into the NcoI and SbfI sites, replacing the nucleotides encoding the Pet passenger but enabling fusion at the C terminus with the Pet AC domain and β-barrel. In addition, to facilitate purification, a polyhistidine tag was incorporated at the N terminus of IrmA, and this section was preceded by the PelB signal sequence to enable translocation across the inner membrane (Fig. 1B). The final construct was termed pET22b-IrmA-S (see Fig. S1 in the supplemental material). The resulting plasmid encodes the fusion of Pet AT and IrmA under the control of an isopropyl-β-d-thiogalactopyranoside (IPTG)-inducible T7 promoter. As the fusion protein is secreted, the PelB signal sequence is removed by the Sec pathway during translocation (Fig. 1C). Then, the β-barrel is inserted into the outer membrane by the BAM complex and IrmA and fused AC are secreted via the β-barrel. Once the protein is surface localized, a proteolytic cleavage event occurs within the β-barrel releasing the Pet AC domain fused to the C terminus of IrmA; we have termed this secreted protein IrmA-S. IrmA-S contains a 1.4-kDa six histidine tag and linker, 14.2 kDa of IrmA (native IrmA without the Sec signal sequence), and the 14.2-kDa Pet AC domain. The final molecular mass of IrmA-S is predicted to be 29.8 kDa, which is a figure that agrees with the observed mass (Fig. 1B).

The cysteine residues present in the antigen IrmA are separated by 60 residues, but disulfide-bonded loops of over 20 residues were shown to be incompatible with the Pet AT translocation though the β-barrel domain (18). Considering this finding, the plasmid pET22b-IrmA-S was transformed into the host strain E. coli BL21*DE3 *dsbA*::*aph*, which is a strain lacking DsbA that is required for cysteine bond formation in the bacterial periplasm, and protein expression was induced with IPTG during growth in lysogeny broth. Proteins secreted into the extracellular milieu were precipitated by the addition of trichloroacetic acid and were analyzed by SDS-PAGE separation followed by Coomassie staining or Western blotting using anti-His tag antibodies (Fig. 1D and E, respectively). Coomassie staining revealed that a single 35-kDa band was in the culture fluid. Western blotting confirmed that this band contained a His tag. Therefore, this band was presumed to be IrmA-S despite the difference in expected mass of 29.8 kDa and the observed mass of approximately 35 kDa after separation by SDS-PAGE. The suspected IrmA-S band was excised and digested by incubation with trypsin. The resulting peptides were analyzed by liquid chromatography-tandem mass spectrometry, and peptides corresponding to both the Pet AT domain and IrmA were identified (see Table S1 in the supplemental material). Furthermore, the identity of IrmA-S was confirmed using liquid chromatography-tandem mass spectrometry without trypsin digestion. The measured molecular mass of IrmA-S was 29,849.46 Da. This result was consistent with the predicted theoretical molecular mass of IrmA-S of 29,841 Da (see Fig. S2 in the supplemental material). Additionally, IrmA-S accounted for more than 90% of the total protein present within the culture medium (Fig. 1D). Thus, IrmA-S was secreted via the Pet AT platform and constituted the majority of the proteins present in the extracellular milieu.

### Purification of secreted IrmA.

**FIG 2:**
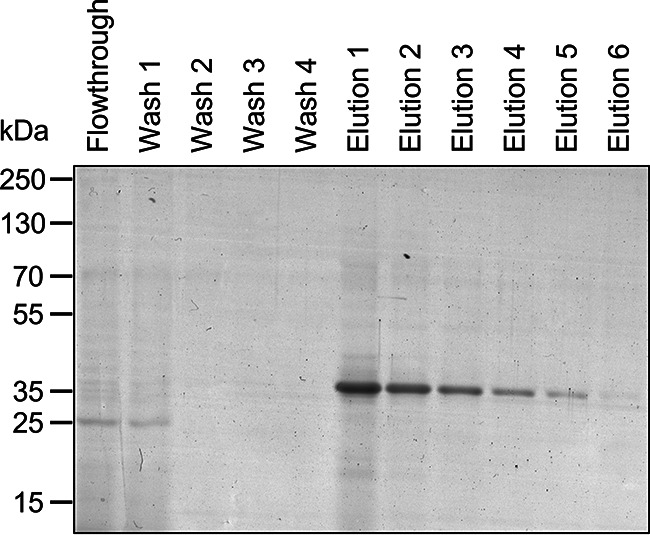
Purification of IrmA-S from the culture supernatant. The culture medium of E. coli BL21*DE3 *dsbA::aph* harboring the plasmid pET22b-IrmA-S was harvested. The culture medium was concentrated by membrane ultrafiltration and dialyzes against the binding buffer prior to incubation with the magnetic beads for purification. IrmA-S was eluted with imidazole.

Having verified that the Pet AT platform could be used to produce IrmA-S as a secreted protein, we sought to determine a method to purify the protein to homogeneity. As IrmA-S was a substantial component of the culture supernatant, we predicted that a single purification step would be adequate for protein purification to homogeneity. Accordingly, an E. coli BL21*DE3 *dsbA*::*aph* strain harboring the pET22b-IrmA-S plasmid was grown in 2 L of lysogeny broth (LB). Cells were harvested by centrifugation, and the culture medium containing IrmA-S was isolated. The culture supernatant fraction was concentrated by membrane ultrafiltration and dialysis against the binding buffer. The concentrated culture medium was incubated with cobalt magnetic beads, and subsequently, IrmA-S was eluted with imidazole. Fractions from the purification were analyzed by SDS-PAGE (Fig. 2). A band corresponding to IrmA-S was detected in the eluted fractions but not in the flowthrough fractions or washes. Therefore, IrmA-S was purified successfully from the extracellular milieu in a single purification step.

### Characterization of secreted IrmA.

**FIG 3:**
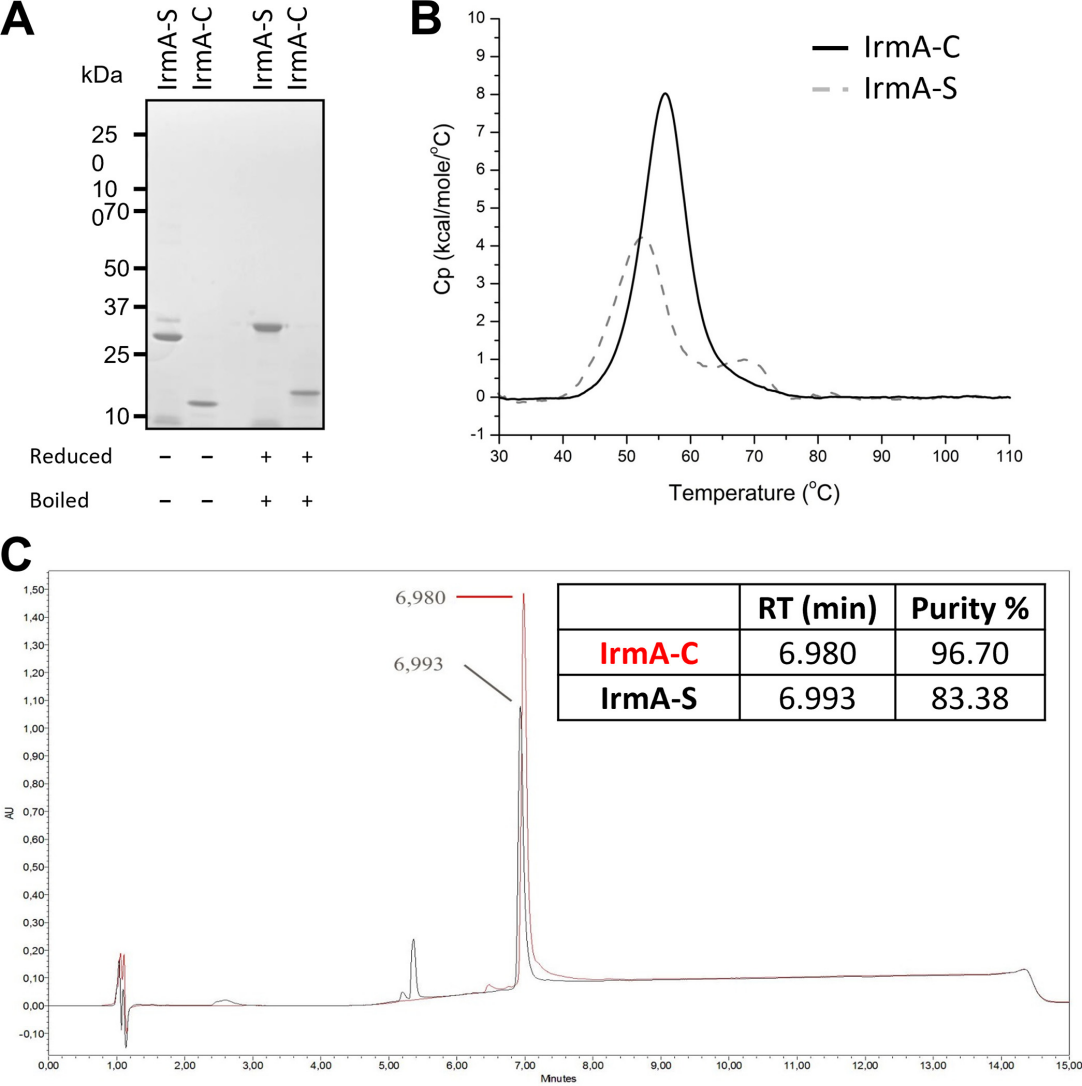
Comparison of IrmA-C and IrmA-S. (A) IrmA-S and IrmA-C were analyzed by SDS-PAGE with or without heating and reduction by DTT. (B) IrmA-S and IrmA-C were analyzed by differential scanning calorimetry to investigate their folding and thermal stability. Scans were recorded, and the spectra are reported in a solid line for IrmA-C and dashed line for IrmA-S. (C) IrmA-S and IrmA-C were analyzed by reverse-phase chromatography.

Purified IrmA-S was compared with IrmA-C to check that the two proteins produced with different systems were biochemically similar. IrmA-S and IrmA-C were first analyzed by SDS-PAGE under reducing and nonreducing conditions (Fig. 3A). Most of IrmA-S and IrmA-C migrated as a single protein band when samples were reduced and boiled. There were two bands in the unreduced IrmA-S sample. Approximately 85% of the nonreduced and unboiled IrmA-S migrated faster than the reduced and boiled samples. A similar phenomenon was noted for IrmA-C indicating that the aberrant migration is due to the behavior of IrmA rather than due to the Pet AC domain. Despite the size difference, both IrmA-S and IrmA-C migrated as monomers in the presence of SDS (Fig. 3A).

The T5SS passenger is usually translocated through its cognate β-barrel domain in an unfolded structure (8). The passenger folds after translocation, and it has been proposed that its folding could be the driving force for the translocation (19). Passenger folding has been shown to happen only after translocation (20). The fold of IrmA-S was compared with that of IrmA-C by differential scanning calorimetry (Fig. 3B). A transition was registered at a melting temperature of 56.1°C for IrmA-C and 52.8°C for IrmA-S. This transition was the transition of the IrmA domain that the two proteins share. A second transition at 68.4°C was detected for IrmA-S and was likely related to the presence of 115 amino acids of the Pet autochaperone domain which is absent in IrmA-C. A biphasic transition for proteins containing two domains has been observed previously and had been noted previously for AT passengers where the AC domain remained covalently attached (21–24). The similarity of the IrmA-C and lower IrmA-S transition temperatures confirms that both share the IrmA protein structure. Furthermore, IrmA-S and IrmA-C were analyzed by reverse-phase chromatography. IrmA-S and IrmA-C were eluted at the same retention time suggesting that the two proteins had a similar hydrophobicity (Fig. 3C). Therefore, despite the differences in the production and purification method of IrmA-S and IrmA-C, both display similar biophysical properties.

### Clone selection for IrmA-S production.

Having demonstrated that IrmA-S had comparable conformation to IrmA-C, we tested a scaled-up production process to determine whether the Pet AT platform was compatible with the high yields required for industrial-scale recombinant protein production. High-cell-density cultures are used to produce a larger amount of the protein. Fermentation is used routinely to achieve a high-cell-density cultivation of E. coli (25). A nutrient-rich medium was chosen instead of standard LB to support growth at high cell density for the fermentation production of IrmA-S. In addition, bacteria were grown at a lower temperature, namely, at 25°C instead of 30 to 37°C, to lower the cellular stress that occurs when an exogenous protein is overexpressed.

**FIG 4:**
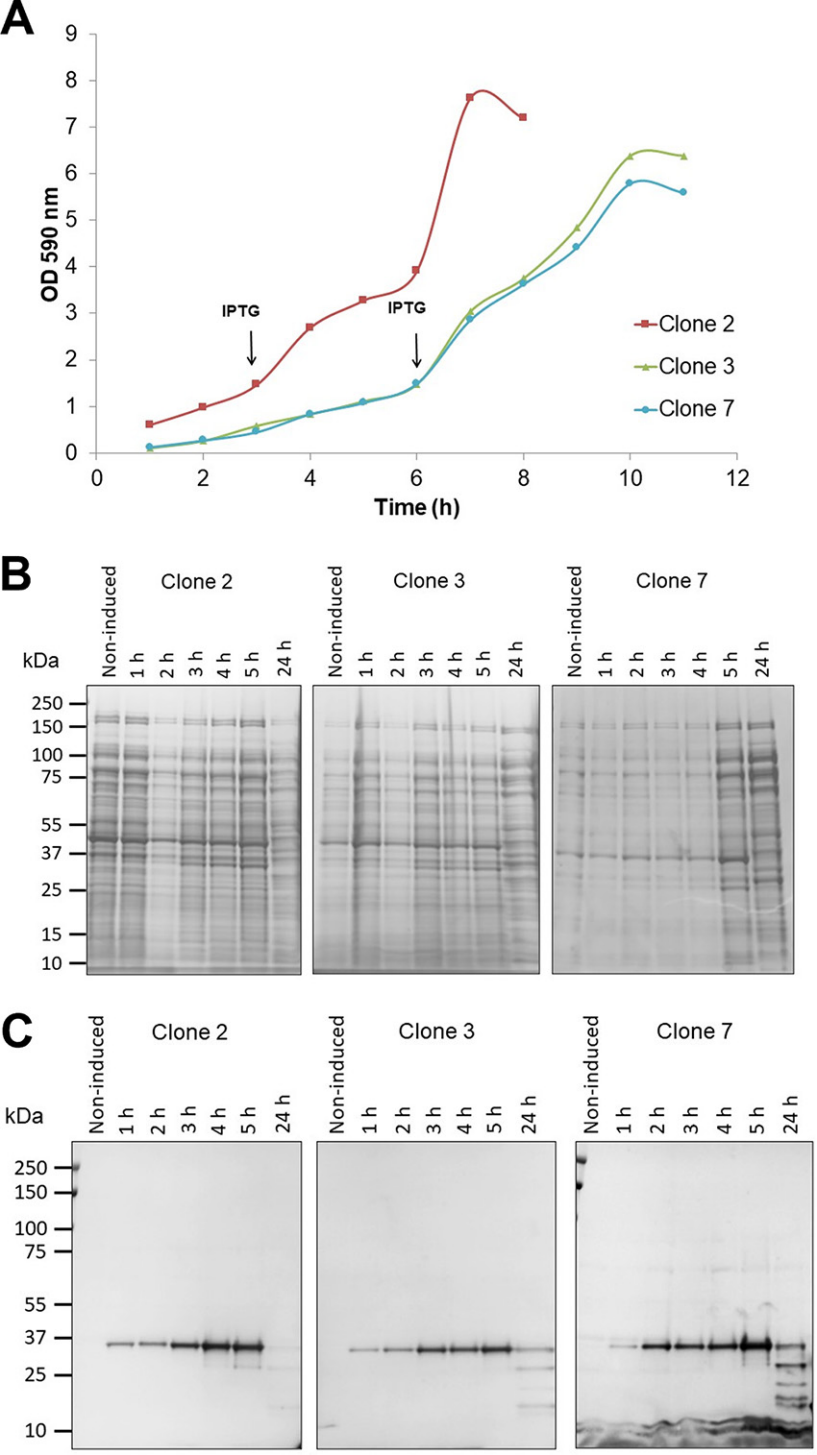
Screening of clones for IrmA-S production. Bacterial clones 2, 3, and 7 were grown in a 50 mL complex medium. IrmA-S expression was induced with IPTG after the culture reached an OD_590_ of 1. (A) The growth of the clones was monitored with OD_590_ measurements. For 5 h after IPTG induction, the culture medium for each clone was harvested and the proteins were precipitated in the presence of trichloroacetic acid. Precipitated protein samples were separated by SDS-PAGE and stained with Coomassie (B) or analyzed by immunoblotting with a α-His antibody (C).

As there could be variations between transformants, the pET22b-IrmA-S plasmid was transformed into E. coli BL21*DE3 *dsbA*::*aph* and 8 clones were screened for IrmA-S accumulation. Three clones were selected for further analysis, namely, clones 2, 3, and 7. These clones were grown in 50 mL of complex medium. IrmA-S production was induced with 0.5 mM IPTG. The growth of the bacteria was assessed by measuring culture optical density at 590 nm (OD_590_) (Fig. 4A). Clone 2 grew more rapidly than clones 3 and 7, which were similar. Samples of culture medium were taken every hour until 5 h postinduction and again at 24 h postinduction. Proteins in the culture medium were precipitated by trichloroacetic acid, separated by SDS-PAGE, and stained with Coomassie (Fig. 4B) or subjected to immunoblotting with anti-His antibodies (Fig. 4C). SDS-PAGE analysis revealed that protein impurities were present in the culture medium at all time points analyzed. This phenomenon was not observed when IrmA-S was produced from small-scale LB cultures (Fig. 1D). Clone 2 was shown to have the fastest growth and comparable IrmA-S production at earlier time points (Fig. 4C). For this reason, it was chosen for further study.

### Scale-up of IrmA production.

**FIG 5:**
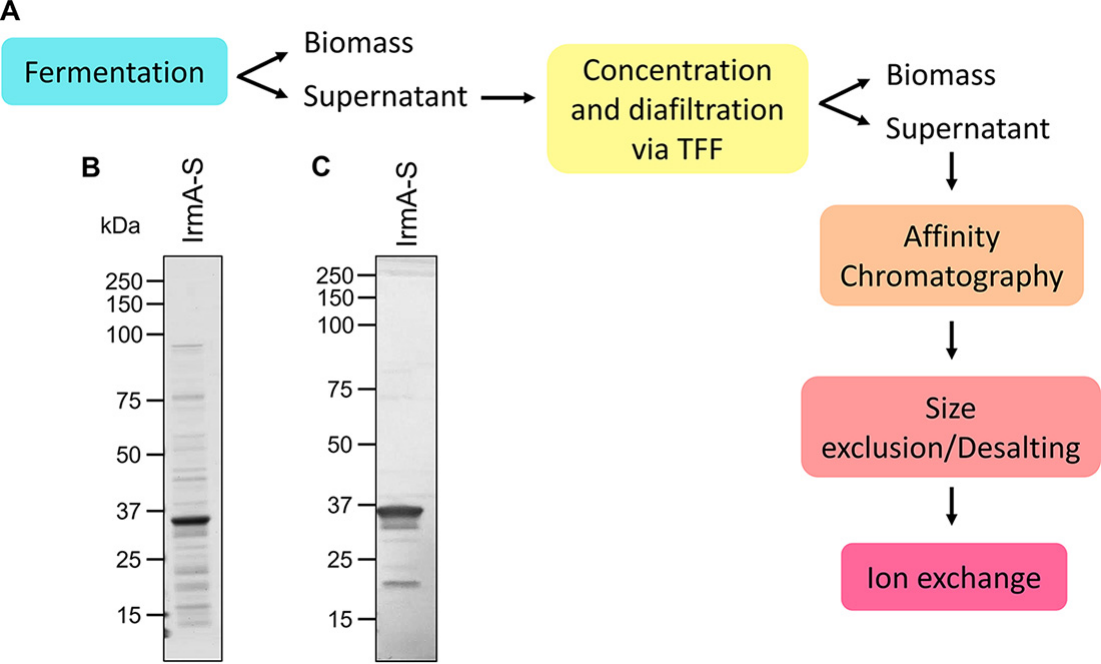
Scaled-up production process for IrmA-S. (A) A schematic of the production process for IrmA-S. After fermentation of the isolated clone, the culture medium was separated and concentrated via tangential flow filtration (TFF). The concentrated culture medium was purified by Ni^2+^ affinity chromatography. The sample was desalted by size exclusion chromatography to remove the imidazole in the eluted fractions. Finally, the antigen was purified by ion-exchange chromatography. A culture of clone 2 was grown in a batch fermenter. IrmA-S accumulation in the culture medium was induced for 3 h with IPTG. The culture supernatant was isolated and concentrated 20-fold by tangential flow filtration. The IrmA-S was purified from the concentrated culture medium by affinity chromatography and ion-exchange chromatography. The purified antigen was analyzed by SDS-PAGE and stained with Coomassie (B) or analyzed by immunoblotting with α-His antibody (C).

Having demonstrated that IrmA-S could be produced in high-density cultures, we next sought to investigate production in scaled-up conditions. A complex medium was used to support bacterial growth to high cell density. A 4-L culture was grown at a fixed temperature of 25°C. IrmA-S production was induced with 0.5 mM IPTG for 3 h. Cells were harvested by centrifugation, and the culture medium was isolated. Due to the presence of process impurities in the culture medium (Fig. 4B), several downstream processing steps were included. A scheme of the process used is shown in Fig. 5A. The culture medium was concentrated by tangential flow filtration to reduce the sample volume and remove impurities. The use of a membrane with a 10-kDa cutoff allowed the isolation of IrmA-S in the retentate. However, many more protein impurities were present in the supernatant fractions from these experiments than those in the small-scale expression experiments highlighted earlier (Fig. 1). These impurities are likely derived from cell lysis, which is a well-known phenomenon in large-scale recombinant protein production (26). Thus, the concentrated culture medium was applied to an Ni^2+^ affinity column. After a wash step, IrmA-S was eluted with imidazole. Imidazole was removed from the eluted fractions by buffer exchange chromatography using a HiPrep desalting column. The resulting IrmA-S-enriched fraction was applied to a Q Sepharose column. After a wash step, the antigen was eluted with NaCl. The purified fraction was separated by SDS-PAGE and analyzed by Coomassie staining (Fig. 5B) or immunoblotting (Fig. 5C). Samples were retained from each stage of the process so that the yield of IrmA-S could be assessed (see Table S2 in the supplemental material). After 3 h of induction, the amount of IrmA-S produced in the culture medium was 29.3 mg/L (Table 1). Despite the further purification steps required for the batch fermentation compared with those for the small-scale purification, a large amount of IrmA-S was isolated. Therefore, the Pet AT platform is suitable for batch fermentation resulting in high yields of secreted soluble protein.

**TABLE 1:** Accumulation of IrmA-S in the culture supernatant

Time postinduction (h)	Amt of IrmA-S secreted into the culture supernatant (mg/L)
1	6.38
2	16.61
3	29.25

## DISCUSSION

Many new biopharmaceuticals have been approved by FDA, including hormones, growth factors, antibodies, vaccines, fusion proteins, and therapeutic enzymes (27). Three hundred and forty-seven biopharmaceuticals had been approved by July 2019, of which 94 were produced in E. coli (28). Sales figures for these drugs are growing, which in turn is increasing competition due to the advent of the first biosimilars on the market. Upstream and downstream processing procedures contribute significantly to the final price of the drug itself, and it has been estimated that the cost of downstream processing alone could account for up to 80% of the total cost of the final drug manufacturing. It has therefore become clear that new systems for effective, low-cost recombinant protein production are required, and a need for simplification in such processes has been emphasized (27). The Pet AT platform was proposed as a possible method to achieve these goals (3).

The standardization, optimization, and reproducibility of protein production are crucial issues in the attempt to produce biopharmaceuticals at a lower cost using bacterial systems. Typically, heterologous proteins are produced in the cytoplasm of the bacterial cell. Cytoplasmic protein production can result in the formation of inclusion bodies or a reduction in biomass due to the production of a toxic protein. Proteins in inclusion bodies must be refolded and may not fold into their native structure. Additionally, the cytoplasmic content of cells must be released by lysis, which may affect protein folding and result in product contamination. Furthermore, the desired protein must now be isolated from the lysate, potentially resulting in contamination by autologous proteins and requiring further purification steps and equipment. These steps are costly and laborious, and each additional step results in product loss. Thus, a possible way to increase production efficiency of an exogenous protein is by targeting the protein to the culture medium in which purification of the protein would be more cost-effective. Currently, IrmA is purified from bacterial inclusion bodies. Thus, the production process is not cost-effective as it requires refolding. Here, the Pet AT platform is an advantageous alternative. The results obtained for the small-scale production of IrmA-S with the Pet AT platform were promising. IrmA-S accounted for more than 90% of the culture supernatant sample (Fig. 1D). IrmA-S present in the supernatant was soluble and did not require a refolding step. Thus, the purification of IrmA-S in a single step directly from the culture supernatant is possible.

Prior to this study, the possibility to scale up a production process based on the Pet AT platform through fermentation had not been explored. Having verified the ability of the Pet AT platform to produce IrmA-S at the laboratory scale, we wanted to demonstrate that the system could be scaled up to obtain high protein yields. We demonstrated that the Pet AT platform could be scaled up and was compatible with culturing conditions, such as batch fermentation. A yield of 29.3 mg/L of IrmA-S in the culture supernatant is very promising. This small-scale study demonstrated that the production of a difficult protein using the Pet AT platform was feasible. It was notable that while IrmA-S represented the majority of protein in the secreted fraction from the initial small-scale experiments, it was a lesser component of the proteins recovered from the supernatant fraction after the scale-up. The optimization of the fermentation process, to increase yields and decrease impurities, has the potential to result in a highly cost-efficient process.

While we have demonstrated the potential for high-yield protein recovery from the supernatant fraction, the IrmA-S construct retains the AC domain. The retention of the AC domain on any heterologous recombinant protein would present challenges to the use of the protein as a vaccine candidate or in a therapeutic setting. Thus, methods to remove the AC domain after purification need to be considered. Previous approaches have included the incorporation of TEV and other protease recognition sites into the target protein followed by an additional round of purification to separate the cleaved components. Such proteolytic sites have been engineered previously into AT to affect the release of the heterologous protein from autodisplay systems, suggesting that this approach would also be useful in this setting (12).

While the AT platform has been demonstrated to produce heterologous proteins, it is not universally amenable to the production of all proteins. Previous observations demonstrated that the presence of disulfide bonds and the presence of structured elements within the passengers influence the efficiency of secretion across the outer membrane. As IrmA possesses two cysteines that are known to form a disulfide bond, we elected to produce both IrmA-S and IrmA-C in a Δ*dsbA* mutant; several studies have shown that the disruptive presence of disulfide bonds can be alleviated by using expression strains lacking the disulfide bond isomerase DsbA or by growth in medium containing reducing agents such as dithiothreitol (DTT) (3, 18, 29). IrmA-S and IrmA-C both exhibited a size shift on boiling, which could be consistent with the reduction of the IrmA disulfide bridge in the reduced and boiled samples (14). However, as disulfide bonds rarely form in the oxidizing environment of the cytoplasm, such that IrmA-C is unlikely to have a disulfide bond, disulfide bonds are often incompatible with transport via the AT system, and both proteins were produced in a DsbA mutant, this size shift is more likely due to changes in the conformation of IrmA itself. The heat modifiability of bacterial outer membrane β-barrel proteins has been reported, where aberrant migration is observed upon boiling of the protein sample prior to separation in SDS-PAGE gels (30, 31). For these proteins, it has been reported that the folded proteins migrate faster than the heat-denatured protein (30). The IrmA structure is composed of β-sheets, which are more resistant to SDS than proteins that contain α-helical folds and typically remain folded in the absence of reducing agents or heat denaturation (32). In this study, IrmA migrates in SDS-PAGE gels as a monomer, although the reported structure revealed a homodimer. While Moriel et al. (14) confirmed that the dimer was present in solution by size exclusion chromatography and blue native-PAGE, the authors also observed a band corresponding to the monomer size when IrmA was separated in a reducing SDS-PAGE gel, suggesting the dimer organization is sensitive to SDS-mediated dissociation.

In conclusion, we describe a system that can secrete a soluble heterologous protein during batch fermentation culture. The Pet AT platform is a versatile single-protein system that could avoid the typical issues of cytoplasmic recombinant protein production at the industrial scale, enabling simple and efficient protein purification from the culture supernatant of fermentation cultures.

## MATERIALS AND METHODS

### Bacterial strains, plasmids, and growth conditions.

E. coli DH5α and E. coli BL21*DE3 were purchased from Invitrogen and Novagen, respectively. E. coli DH5α was used for cloning and plasmid preparation. The pET22b-IrmA-S plasmid was constructed by amplifying the *irmA* gene using primers pET22b-irmA_S_FW (GGCGGCCGCCATGGATCAGGATCAACGTTACATCAG) and pET22b-irmA-S_RV (GGCGGCCCTGCAGAGTTAACGTTTTTTCCGGAAAC), followed by cleavage of the amplified product with NcoI and SbfI and subsequent ligation into pET-Pet which had been digested with NcoI and PstI. The complete sequence of pET22b-IrmA-S is available from FigShare (https://bit.ly/3Wq2qs1). E. coli BL21*DE3 *dsbA*::*aph* was constructed by P1 transduction from E. coli TOP10 *dsbA*::*aph* (18). E. coli BL21*DE3 *dsbA*::*aph* was cultured routinely in lysogeny broth (LB), 10 g/L tryptone, 5 g/L yeast extract, and 5 g/L NaCl and used for recombinant protein production. For batch fermentation E. coli BL21*DE3 *dsbA*::*aph* was cultured in a complex medium composed of 45 g/L yeast extract, 10 g/L glycerol, 16 g/L K_2_PO_4_, and 4 g/L KH_2_PO_4_ under scaled-up conditions. If required, culture media were supplemented with the appropriate antibiotics and inducers as required, as follows: ampicillin (100 μg/mL), kanamycin (25 μg/mL), and IPTG (0.5 mM).

### SDS-PAGE and immunoblotting.

Sample buffer, Laemmli 2× concentrate (Sigma) and 2 μL of 1 M DTT (Invitrogen) was mixed with protein samples prior to incubation at 100°C for 5 min. When a nonreducing and nondenaturing analysis was required, samples were treated as specified before but by omitting DTT and heating. Samples were loaded onto a precasted 4 to 12% SDS-PAGE protein gel (Invitrogen) with PageRuler plus prestained protein ladder (Thermo Scientific). Proteins were detected using brilliant blue G-colloidal concentrate, electrophoresis reagent for protein detection (Sigma) as per the manufacturer’s instructions. Specific proteins were detected by immunoblotting. In brief, proteins were separated on an SDS-PAGE gel and transferred to nitrocellulose using the iBlot gel transfer system (Life Technologies). The membrane was incubated with a blotting solution (5% [wt/vol] skimmed milk powder and phosphate-buffered saline [PBS] supplemented with 0.1% [vol/vol] Tween 20). The membrane was washed three times with PBS supplemented with 0.1% (vol/vol) Tween 20 (PBST) and incubated with a dilution of the appropriate primary antibody in blotting solution. A 1:5,000 dilution of a mouse monoclonal antibody raised against a polyhistidine tag was used. The membrane was washed three times with PBST prior to incubation with a dilution of the appropriate secondary antibody in blotting solution. A 1:30,000 dilution of anti-mouse alkaline phosphatase-conjugated antibodies (Sigma) was used as secondary antibodies. The membrane was washed three times with PBST prior to protein detection with BCIP/NBT-purple liquid substrate system for membranes (Thermo Fisher) or the Opti-4CN substrate kit (BIORAD) according to the manufacturer’s instructions.

### Culture supernatant protein precipitation.

Culture volumes were adjusted according to the OD_590_ of the cultures. Cells were removed by centrifugation at 3,724 × *g* for 20 min at 4°C. Culture supernatants were filtered through 0.22-μm filters (Millipore), and a 1:10 (vol/vol) dilution of ice-cold 100% trichloroacetic acid was added to each sample. Samples were incubated on ice for 45 min, and precipitated proteins were spun down at 15,366 × *g* for 45 min at 4°C. Supernatants were discarded, and pellets were resuspended in 1 mL of ice-cold 100% acetone. Samples were centrifuged at 18,407 × *g* for 1 h at 4°C to remove acetone. The resulting pellets were resuspended in 100 μL of sample buffer, Laemmli 2× concentrate (Sigma) supplemented with saturated Tris solution and 1 M DTT (Invitrogen) prior to analysis by SDS-PAGE and immunoblotting.

### Small-scale purification of IrmA.

Cultures were grown at temperatures from 30 to 37°C for small-scale IrmA-S production. An aliquot of 150 mL of culture supernatant was concentrated to 3 to 4 mL using VivaSpin columns (Sartorius) with a 10-kDa cutoff. The concentrated culture supernatant was dialyzed overnight at 4°C against binding buffer (50 mM Na_2_HPO_4_ [pH 8], 300 mM NaCl, and 0.01% Tween 20). Samples were incubated overnight with 150 μL of Dynabeads (Life Technologies) at 4°C. In the morning, Dynabeads were washed 4 times with 1 mL of binding buffer and then six times with 100 μL of elution buffer (50 mM Na2HPO4 [pH 8], 300 mM NaCl, 0.01% Tween 20, and 300 mM imidazole).

### Batch fermentation.

A 50-mL culture of the selected strain was inoculated from glycerol stock. The medium used was composed of 45 g/L yeast extract, 10 g/L glycerol, 16 g/L K_2_PO_4_, and 4 g/L KH_2_PO_4_ supplemented with 100 μg/mL ampicillin. A total of 100 mL of broth was inoculated to an OD_590_ of 0.2 with an overnight-aerated culture and grown in a shaking incubator at 25°C at 180 rpm. A 7-L autoclavable ADI bioreactor (Applikon) with a working volume of 4 L containing the medium specified above was inoculated to an OD_590_ of 0.05. Foam formation was prevented with PPG at a concentration of 0.75 mL/L, temperature was set at 25°C, pH was maintained at 7.2 with 4 M NaOH and 2 M H_3_PO_4_, and dissolved oxygen concentration and stirrer speed were controlled with the Bioxpert software (Applikon). The fermenter culture was induced at an OD_600_ of 12.5 for 3 h with 0.5 mM IPTG. Simultaneously, 30 g/L glycerol, 0.25 g/L MgSO_4_, and 50 mg/L ampicillin were added. Bacteria were pelleted by centrifugation at 12,000 × *g* for 1 h at 4°C to remove cell debris and particulates. Subsequently, the supernatant fraction was filter sterilized through a 0.22-μm filtering unit (Corning) and kept at 4°C prior to further analysis.

### Scaled-up purification of IrmA.

Two liters of fermentation supernatant were concentrated 20-fold at 4°C by tangential flow filtration using a Sartocon Slice 200 device (200-cm^2^ filter area; 10-kDa cutoff; Hydrosart membrane; Sartorius). The retentate containing IrmA-S was then diafiltrated against 20 volumes of PBS. During the diafiltration step, the retentate volume was kept constant, the input pressure was maintained at 1.8 to 2.0 bar, and transmembrane pressure was maintained at 1.1 to 1.2 bar. The retentate was collected for further purification of IrmA-S.

The tangential flow filtration retentate was purified by affinity chromatography using an Äkata purifier 10 system (GE Healthcare) and a 1-mL HisTrap high-performance (HP) column (GE Healthcare). Protein absorbance at 214, 260, and 280 nm was monitored. The mobile phase (binding buffer HP) was composed of 50 mM Na_2_HPO_4_ (pH 8) and 300 mM NaCl and was used to equilibrate the column with a 1-mL/min flow rate in 10 column volumes. The culture supernatant was loaded onto the column at a 1-mL/min flow rate. The column was washed with 5 column volumes of binding buffer HP. IrmA-S was eluted with elution buffer HP (50 mM Na_2_HPO_4_ [pH 8], 300 mM NaCl, and 400 mM imidazole) at a 1-mL/min flow rate in 10 column volumes. A total of 1-mL fractions were collected at every purification step for further analysis. If required, the fractions containing IrmA-S were pooled and dialyzed against 2 L of binding buffer HP overnight at 4°C. Proteins were quantified by the micro-bicinchoninic acid (BCA) assay using the micro-BCA protein assay kit (Thermo Scientific). Affinity chromatography fractions containing the antigen IrmA-S were combined. Salt was removed by buffer exchange chromatography. A Sephadex G-25 HiPrep 26/10 desalting column (GE Healthcare) was equilibrated with 50 mM Tris-HCl (pH 8.0) at a 4-mL/min flow rate. The sample was loaded on the column and eluted isocratically at a 4-mL/min flow rate for 1.5 column volumes. Fractions of the void volume were pooled and subjected to further purification by ion-exchange chromatography. A HiTrap Q Sepharose FF column (GE Healthcare) was used. Protein absorbance at 215, 260, and 280 nm was detected. The column was equilibrated with 50 mM Tris-HCl (pH 8.0; binding buffer) at a 1-mL/min flow rate. Fractions were loaded on the column with a 1-mL/min flow rate. The column was washed with 5 column volumes of binding buffer. IrmA-S was eluted with a linear gradient from 0% to 100% of 500 mM NaCl in 20 column volumes of binding buffer. Next, 1-mL fractions were collected and analyzed by SDS-PAGE to determine the fractions containing IrmA-S.

### Differential scanning calorimetry.

A MicroCal VP-capillary differential scanning calorimetry (DSC) instrument (GE Healthcare) with an integrated autosampler was used for DSC. In brief, DSC scans were in the temperature range of 10 to 130°C with a thermal ramping of 150°C per h and a 5-s filter period. Samples were exchanged in PBS (pH 7.4). For each sample, 500 μL at a concentration of 12 μM (estimated by Nanodrop) was analyzed; the samples were transferred to a 96-well plate and left in the instrument autosampler at 5°C until analysis. A 30 μM lysozyme solution was analyzed at the beginning and at the end of the set of experiments to confirm a high degree of reproducibility of the data collected (melting temperature [*T_m_*] = 71.2 ± 0.1). Data were analyzed using the Origin 7 software (OriginLab), subtracting the data recorded for a sample containing only buffer to the reference data.

### Reverse-phase high-pressure liquid chromatography.

IrmA-C and IrmA-S were analyzed by reverse-phase high-pressure liquid chromatography (RP-HPLC) with the Acquity-ultraperformance liquid chromatography (UPLC) system (Waters). Samples were analyzed on a BEH C4 column (300 Å; 2.1 by 150 mm; 1.7-μm particles size; Acuity UPLC protein). The mobile phase was composed of buffer A (0.1% trifluoroacetic acid [TFA]) and buffer B (0.1% TFA and 90% acetonitrile). Samples were eluted at a flow rate of 4 mL/min in three steps, as follows: with 97.7% buffer A and 2.3% buffer B for 2.5 min; with 0% buffer A and 100% buffer B for 10 min; and finally, with 97.7% buffer A and 2.3% buffer B for 2.5 min. Protein absorbance at 215, 260, and 280 nm was measured.
