# Supplementary material for: Efficient Autotransporter-Mediated Extracellular Secretion of a Heterologous Recombinant Protein by Escherichia coli
Source: Microbiol Spectr. 2023 Apr 10;11(3):e03594-22. doi: 10.1128/spectrum.03594-22 (PMC10269718; doi:10.1128/spectrum.03594-22)
Supplement: Supplemental file 1 — Fig. S1 and S2 and Tables S1 and S2. Download spectrum.03594-22-s0001.pdf, PDF file, 0.2 MB [file spectrum.03594-22-s0001.pdf]

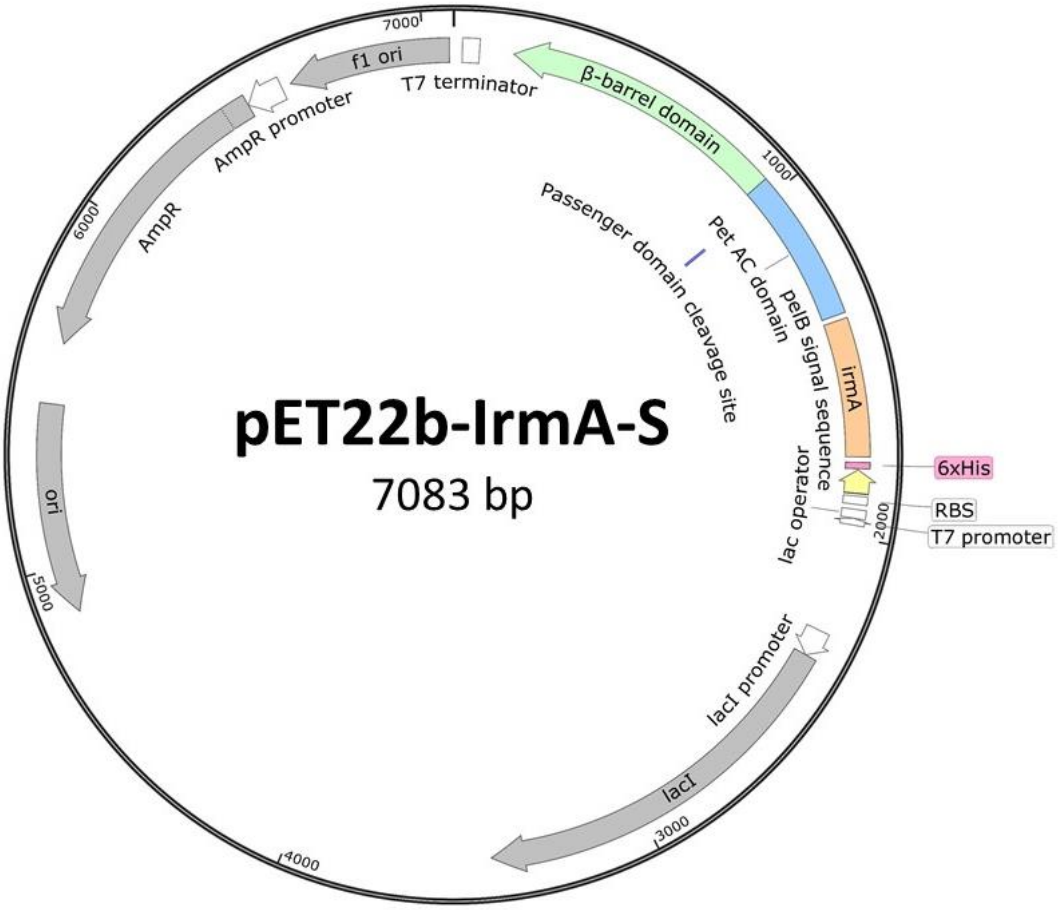

Figure S1: Plasmid map of pET22b-IrmA-S. The *irmA* gene sequence was amplified from a GSK plasmid and then subcloned into the NcoI and SbfI restriction sites in the pET22b plasmid that encoded the Pet autotransporter. The *irmA* was fused at the C-terminus to the Pet AC domain and at the N-terminus with a poly histidine tag and PelB signal sequence. The gene is transcribed from a T7 promoter upon IPTG addition. Upon autocatalytic cleavage at the passenger cleavage site, IrmA-S is released from the β-barrel domain. The Pet AC domain and α-helix portion fused to IrmA accounts for 15 kDa, IrmA-S molecular mass was therefore predicted to be 29.8 kDa. The plasmid map was created using SnapGene.

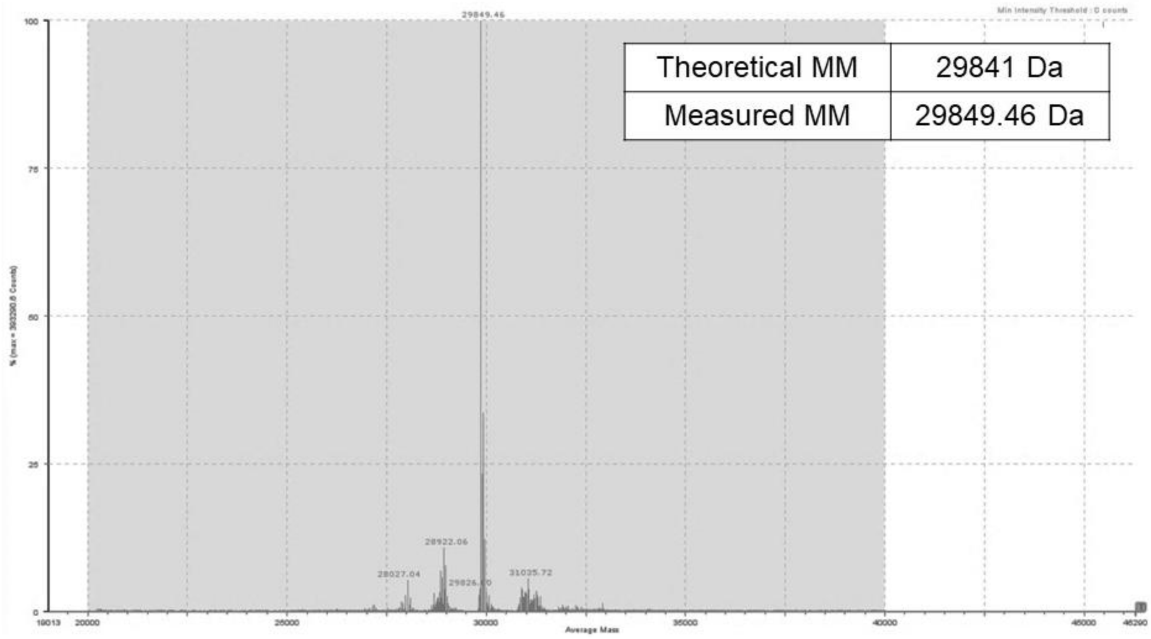

Figure S2: Mass spectrometry analysis of IrmA-S. The purified IrmA-S antigen has been analysed by mass spectrometry to measure the molecular mass of the protein sample. Measured molecular mass was compared with the theoretical value.

Table S1: Peptide score for IrmA-S

Coverage (%)	# Peptides	# AA	Score	Description
8.19	23	1295	661.76	Serine protease (plasmid-encoded toxin Pet) [*Escherichia coli* 042]
75.50	11	151	144.08	Hypothetical protein IrmA [*Escherichia coli* CFT073]

Table S2: Percentage yield from each processing step for IrmA-S.

Purification step	Yield (%)
TFF	72
Affinity	100
Desalting/Size exclusion	91
Ion Exchange	33
